# Fun and Physic

**Published:** 1871-06

**Authors:** 


### "FUN AND PHYSIC,"

Is a book containing twelve hundred sayings, uttered in the pages of THE JOURNAL OF HEALTH during the last eighteen years, in sentences of a line, or two or three, which one can read, then lay down the book and think about it: small enough to be carried in the pocket, and plain enough to be comprehended without the trouble of thinking. The volume has already made its way to Europe, and a young lady correspondent from Florence writes, "The title takes amazingly. Quotations are served up at every meal; there is a quotation ready to be applied on nearly every subject that comes up. What Dr. Hall says has become quite a joke. The Scotch minister, whenever he wants us to do anything, makes up something to suit himself, and then says Dr. Hall says so." The appreciation of the book at home as well as abroad makes one of many bright spots of a lifetime.

